# Effect of Calcium Ion Removal, Ionic Strength, and Temperature on the Conformation Change in Calmodulin Protein at Physiological pH

**DOI:** 10.1155/2014/329703

**Published:** 2014-12-09

**Authors:** Sunita Negi

**Affiliations:** Cluster Innovation Centre (CIC), University of Delhi, Delhi 110007, India

## Abstract

The response of the calmodulin (CaM) protein as a function of calcium ion removal, ionic strength, and temperature at physiological pH condition was investigated using classical molecular dynamics simulations. Changing the ionic strength and temperature came out to be two of the possible routes for observing a conformation change in the protein. This behavior is similar to the conformation change observed in our previous study where a change in the pH was observed to trigger a conformation change in this protein. In the present study, as the calcium ions are removed from the protein, the protein is observed to acquire more flexibility. This flexibility is observed to be more prominent at a higher ionic strength. At a lower ionic strength of 150 mM with all the four calcium ions intact, the N- and C-lobes are observed to come close to a distance of 30 Å starting from an initial separation distance of 48 Å. This conformation change is observed to take place around 50 ns in a simulation of 100 ns. As a second parameter, temperature is observed to play a key role in the conformation change of the protein. With an increase in the temperature, the protein is observed to acquire a more compact form with the formation of different salt bridges between the residues of the N- and the C-lobes. The salt bridge formation leads to an overall lowering of the energy of the protein thus favoring the bending of the two lobes towards each other. The improper and dihedral terms show a significant shift thus leading to a more compact form on increasing the temperature. Another set of simulations is also performed at an increased temperature of 500 K to verify the reproducibility of the results. Thus a set of three possible alterations in the environmental conditions of the protein CaM are studied, with two of them giving rise to a conformation change and one adding flexibility to the protein.

## 1. Introduction

Proteins are one of the most important parts present inside the eukaryotic cells. One such protein named calmodulin is well known for its key role as a calcium ion carrier inside these cells [1]. This protein helps in various physiological processes inside these cells by activating different enzymes of various types [2]. Mainly it acts as a calcium ion receptor inside the calcium signaling pathways of the cells [1]. The protein is also known for its capability that it can bind to a bulk of other proteins or small organic compounds very well. Structurally CaM can be found in both the free and bound forms as reported in [2, 3]. These forms are defined depending on the calcium ion concentration associated with the protein. Because of its various available forms, this protein is capable of sustaining large conformation changes which depend upon the partner to which it is interacting [1, 3, 4]. These conformation changes would greatly be affected by the various environmental conditions of the protein. It would therefore be interesting to study the effect of these environmental conditions on the possible conformation changes observed in the protein. We therefore study the effect of variations in the environmental conditions such as ionic strength, pH, calcium ion concentration, and temperature on the conformation change of CaM. The environmental conditions would include a vast number of parameters though these sets of conditions are chosen as a first step in this work.

A large number of studies have been performed on CaM because of its important role in different physiological processes. These studies have been used to study the conformation changes as in [5–8]. A number of these studies are performed using molecular dynamics (MD) simulations. Typically these conformation changes are observed on the ns time scale as in [5]. In this study the central helix was observed to bend and eventually unwind near the middle of the linker. In [6], Ca2+ loaded CaM was used to study transitions from an open to a closed state. This transition is also observed to take place on a time scale of ns as in [5]. In most of these studies SPC model is used for simulating the water molecules in the simulations [5, 6]. The open crystal structure of this protein is observed to attain a more compact shape in [7] as well. This change in the shape of the protein gets eventually used in the binding with the target proteins/peptides. The shape or the conformation change of the protein is studied in terms of the orientation between the N- and C-lobes. In [7] this orientation is observed to be slightly different as compared to the earlier studies. The effect of calcium ion removal is also studied in some of the studies as in [8, 9]. Molecular dynamics simulations are used to study the effect of Ca2+ ions dependent conformation change of the protein [8, 9]. In [8] the open to closed structure conformation of CaM is simulated starting with the Ca2+-bound X-ray structure, and a conformation change was obtained after the removal of the Ca2+ ions from the initial crystal structure. These local rearrangements between the two lobes are studied extensively using MD simulations. The lowest root mean square deviation (RMSD) gives an idea about the final stable structure obtained in the simulation and can be compared with the already available experimental data. The removal of calcium ions from the CaM protein is thus observed to play a very significant role in the conformation change of it as the calcium ions are observed to harden the protein structure [9, 10]. In [9, 10] molecular dynamics simulations were used to study the Ca2+ ions dependent conformation change of this protein. Ca2+ ions were observed to play a very significant role in the observed conformation changes by hardening the protein structure. The effect of ionic strength on the conformational multiplicity of Ca2+-CaM is studied in [11]. The tendency of the nonspecific anions to accumulate near the protein surface is observed to increase by lowering the ionic strength.

The other approach to study the conformation changes of the protein is by using different experimental techniques. Methods such as X-ray crystallography, nuclear magnetic resonance (NMR), and fluorescence resonance energy transfer (FRET) have been used to study the conformation changes in the CaM [12–14]. Out of these experimental techniques X-ray crystallography is a “static” method which uses one time snapshot of the protein coordinates whereas the dynamic methods such as NMR and FRET are based on averaging over an ensemble of structures of the protein. These experimental techniques have shown that CaM protein is capable of sampling multiple conformations. Also, it is being shown that these conformation changes would greatly be affected by changing the environmental conditions of the protein. Effects of various environmental conditions such as pH, Ca+2 concentrations, and ionic strength are being reported earlier [12–14] but none of these studies were able to describe a complete set of conditions together that would trigger a conformation change in the protein. Therefore, identifying such a possible set of environmental conditions together would be a possible route to study the different conformation changes that are sampled by the protein.

In our earlier work [15, 16] we studied the effect of varying pH and observed a conformation change in CaM triggered by protonating nine acidic residues of the protein. Four different servers H++ [17, 18], pKd [19, 20], propKa 3.1 [21], and PHEMTO [22, 23] were used to calculate the pKa values of all the titratable groups. Only nine acidic residues 11, 31, 67, 84, 93, 104, 122, 133, and 140 were observed to consistently have upshifted pKa values from the standard values to 5.5 or more. Both the extended and compact forms of the calcium loaded protein were used in this study. Extensive molecular dynamics simulations of two hundred nanoseconds were performed and a conformation change is observed in less than 100 ns. TIP3P model was used for simulating water molecules. The structure thus attained was consistent with the earlier observed structure of fluorescence resonance energy transfer [9] experimental results as well as structures obtained from NMR data [13]. The study suggested that, at high pH, barrier crossing to the compact form of the protein is prevented by the repulsive electrostatic interactions between the two lobes. Thus lowering of pH significantly reduces this barrier height and changes the overall conformational landscape. The key events leading to this conformation shift such as salt bridge and hydrophobic patch formation were also discussed in great detail.

As stated earlier, CaM is capable of sustaining large conformation changes that would depend upon its interacting partner and therefore the environmental conditions would play a major role in this interaction. Therefore, we study the effect of removing calcium ions, changing the ionic strength, and varying temperature on the conformation change of the CaM protein. The environmental conditions studied here would not be the complete possible set but would give a useful insight into the behavior of the protein in different set of environment conditions. MD simulations of 100 ns each are carried out for all the above-mentioned set of conditions. The pH is kept fixed at a physiological value of 7.4 throughout all the simulation sets. The detailed response of the protein is then studied under all the above-mentioned environmental conditions.

## 2. Methods

### 2.1. Calmodulin Structure

**Figure 1:**
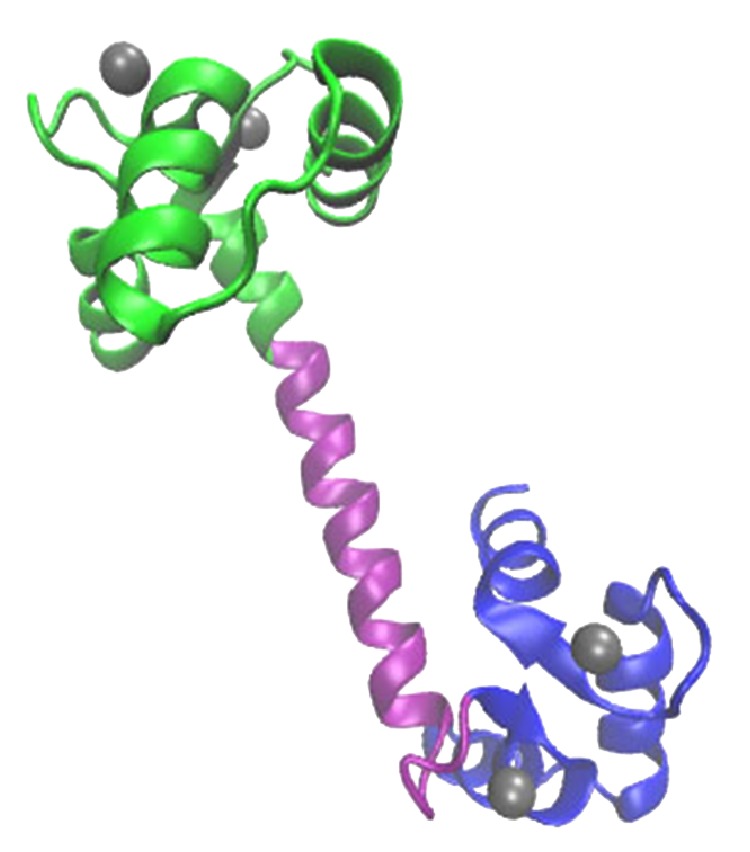
Fully loaded calmodulin (CaM) protein with four calcium ions (shown in grey color); N-lobe shown in green, the linker in purple, and the C-lobe in cyan.

CaM protein consists of 148 amino acids made up of two lobes the N-lobe (residues 1–68) and the C-lobe (residues 92–148). The two lobes are connected with a central linker/helix (residues 69–91). The protein can be observed in two different forms, namely, Apo-CaM which is a form free of calcium ions and holo-CaM which is fully loaded with four calcium ions. Ca2+ ions associated with the protein activate CaM to interact with other proteins/peptides. Fully loaded CaM free of other ligands has two distinct experimentally determined conformations. In the X-ray structures, CaM has an open form with a dumbbell shape containing two domains, joined by an extended flexible linker. This structure can be obtained from the Protein Data Bank (PDB code 3CLN), as shown in Figure 1. The N- and the C-lobes are shown in green and cyan color, respectively. Linker is shown in purple color. Another form of this protein is compact, whereby the central linker is bent (PDB code 1PRW3). Each of the N- and the C-lobes holds two calcium ions each in their respective EF-hand motifs. The coordinating residues in each of the four EF-hands are D20-D22-D24-E31 in loop I, D56-D58-N60-E67 in loop II, D93-D95-N97-E104 in loop III, and D129-D131-D133-E140 in loop IV.

### 2.2. Molecular Dynamics (MD) Simulation Details

In this study we use the extended form of CaM (3CLN) as explained in the section above. Residues 1–5 and 148 are not used in the study as the X-ray data is not available for these residues. Three different sets of simulations are performed with the extended initial 3CLN structure: (a) calcium ions being removed from the 3CLN structure; (b) ionic strength of the solution varied to 150 mM; and (c) the temperature varied to 500 K from an initial value of 300 K. All these sets of simulations are performed for 100 ns each. In the first case, calcium ions are removed from the N-lobe first and thereafter from the C-lobe. In the second case, the ionic strength is varied to 150 mM using the “Autoionize” plugin of the VMD 1.8.7 program [24]. This plugin adds NaCl ions to ionize the solution to a desired value, 150 mM in this case. In the third case, the temperature is varied to 500 K by changing its value in the psf input file of the NAMD program [25]. NAMD package is used to model the dynamics of the protein-water system in all the cases.

We first soak the protein in a water box of at least 10 Å from all directions using the VMD 1.8.7 program with solvate plug-in version 1.2.31. The CharmM27 force field parameters are used for protein and water molecules. Water molecules are described by the TIP3P model. The initial box has dimensions 68 × 84 × 85 Å containing 33830, 33955, and 33955 atoms in the simulation sets 1, 2, and 3, respectively, neutralized by the standard addition of the ions. Long range electrostatic interactions are calculated by the particle mesh Ewald method [26], with a cutoff distance of 12 Å and a switching function at 10 Å. RATTLE algorithm [27] is applied to use a step size of 2 fs in the Verlet algorithm [28].

Temperature control is carried out by Langevin dynamics with a damping coefficient of 5/ps. Pressure control is applied by a Langevin piston. Volumetric fluctuations are preset to be isotropic. The system is run in the NPT ensemble at 1 atm pressure and a temperature of 310 K until volumetric fluctuations are stable to maintain the desired average pressure. The run in the NPT ensemble is then extended to 100 ns for the actual data collection stage. The coordinate sets are saved at an interval of 2 ps for subsequent analysis, leading to a total of 50 000 snapshots during the 100 ns simulation.

Thus three respective sets of simulations are performed to study the behavior of CaM. In the first case, calcium ions are removed one after another from the initial extended 3CLN structure at an ionic strength of physiological value of 200 mM. In the second set, the ionic strength is varied from physiological value to a value of 150 mM keeping the calcium ion concentration to be intact at its original. In the third case, only the temperature is varied to 500 K from an initial value of 300 K keeping both the calcium ion concentration and ionic strength at the original value. The temperature is kept fixed at a value of 300 K in the first two cases and the pH is kept at a physiological value in all the three simulation sets.

## 3. Results

In this section we discuss the results of all our MD simulation sets: (1) calcium ion removal, (2) ionic strength change, and (3) temperature variation case. All significant changes that take place in the form of conformation changes of the protein due to the above-mentioned variations in the environmental conditions are discussed here. The key events which lead to these conformation changes would also be discussed. Root mean square deviation (RMSD) and distance distribution of residues 34–110 are used as a measure to study these conformations of the protein. These distance distributions are calculated from the native structure simulations and give a measure of the compactness of the protein structure.

### 3.1. Effect of Calcium Ion Removal

#### 3.1.1. Root Mean Square Deviations (RMSD)

**Figure 2:**
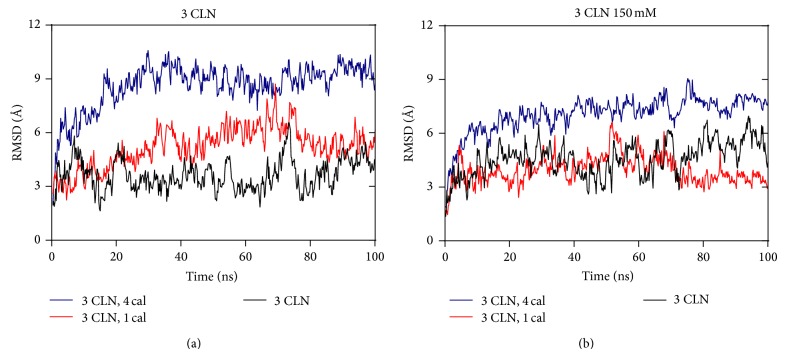
RMSD as a function of time step: (a) ionic strength corresponding to a physiological value; (b) ionic strength equal to 150 mM. In both the cases, RMSD is observed to be higher when all the four calcium ions are removed from the protein.

Calcium ions are removed from the native structure, first from the N- and then from the C-lobes, and the root mean square deviations of the different cases are compared with the original native state as shown in Figure 2. In the first case, which corresponds to an ionic strength of physiological value, the mobility of the protein is observed to increase with the number of calcium ions removed from the protein. The RMSD shifts from an average value of 4 Å to 9 Å as seen in Figure 2(a), when all the four calcium ions are removed. This is consistent with the results of [9, 10] which also report on the effect of calcium ion removal on the stability of CaM. In these studies, the calcium ions were observed to give stiffness to this protein. As seen in Figure 2(a), in all the three sets of simulations, the overall RMSD of the protein gets stabilized during the last 20 ns which indicate a more stable structure during this period. In the second case that is, at low ionic strength of 150 mM, the RMSD shift attains an average value of 7 Å when all the four calcium ions are removed from the protein which is lesser than the shift observed in the previous case. Also, there is no significant change observed in the RMSD on removal of first calcium ion from the native structure. This implies that the calcium ion removal favors more flexibility in the protein structure at its original ionic strength corresponding to physiological value.

#### 3.1.2. Change in the Residues 34–110 Distance Distribution

**Figure 3:**
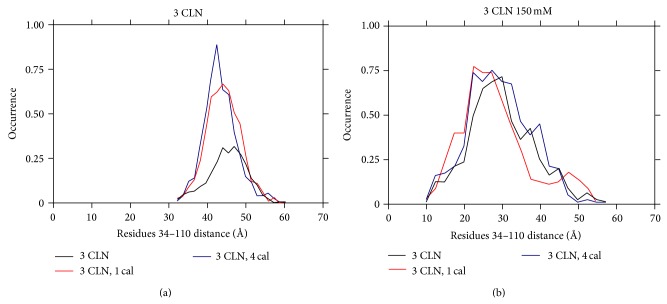
The normalized distance distribution (in Å) measured between residues 34 and 110, observed at the end of 100 ns in (a) ionic strength corresponding to a physiological value and (b) ionic strength equal to 150 mM.

The distance distributions are calculated between the residues 34 and 110, observed at the end of 100 ns as in [12, 15, 16] which gives a measure of the compactness of the protein structure. The respective normalized distance distributions are as seen in Figure 3 with different stages of the calcium ion removal from the protein shown in different colors. The overall distance distributions do not show a significant shift on the removal of calcium ions in each case. Only the distribution peak is observed to acquire a higher value in the case of physiological ionic strength on the removal of calcium ions from the extended form as seen in Figure 3(a). This implies that in this case the calcium ion removal just acts as an attribute to add flexibility to the overall protein structure rather than giving any overall compactness. In the case of lower ionic strength of 150 mM, the normalized distance distribution is not observed to show any significant change. This is in agreement with our earlier result that the calcium ion removal does not make any significant changes in the conformation/flexibility of the protein at a lower ionic strength.

### 3.2. Effect of Change in Ionic Strength

#### 3.2.1. Change in the Residues 34–110 Distance Distribution

**Figure 4:**
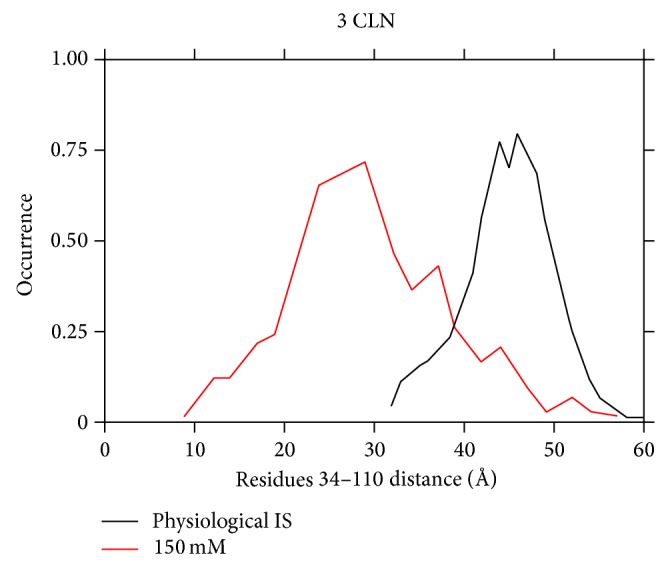
The normalized distance distribution (in Å) measured between residues 34 and 110, observed at the end of 100 ns at ionic strength corresponding to a physiological value and 150 mM. These distance distributions are calculated from the native structure simulations.

The ionic strength is changed to 150 mM using the “Autoionize” plugin in the VMD program as explained in Section 2. The calcium ions are kept intact at their original position in this set of simulations. The CaM protein is observed to change its conformation to a more compact form in this case. This can be inferred from the shift in the normalized distance distributions of residues 34–110 as seen in Figure 4. For this we monitor the distance distribution between the residues numbers 34 and 110 throughout the simulation. A normalized cumulative distance distribution is then calculated which shows a trend similar to what is observed in the lower pH case [15, 16]. These normalized distance distributions are calculated at the end of the 100 ns simulations of the native structure. The distribution here is observed to shift from a mean value of 48 Å to 30 Å on changing the ionic strength from physiological value to a value of 150 mM which implies a compact form of the protein.

#### 3.2.2. Root Mean Square Deviations (RMSD)

**Figure 5:**
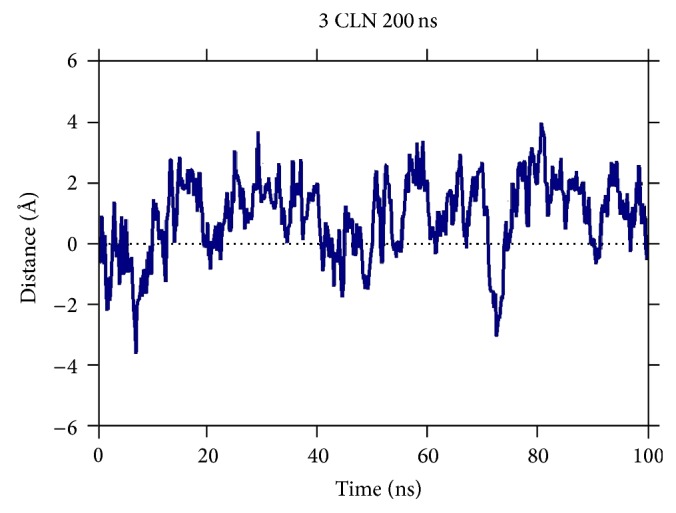
Difference in the RMSD values of the two cases: ionic strength corresponding to a physiological value and corresponding to 150 mM.

The conformation change observed in the protein can also be inferred from the difference in the RMSD values of the two cases, that is, ionic strength corresponding to physiological value and 150 mM as seen in Figure 5. The RMSD difference shows a significant difference ranging from −4 Å to 4 Å. This implies that the low ionic strength paves the pathways for the two lobes to come close to each other which lead to a more compact form as compared to the extended native configuration. This is in accordance with the case observed in [15, 16] where a change in the pH to a lower value also showed similar results. This change is observed because of the loss of electrostatic interactions at a lower pH or lower ionic strength of the solution which gives good opportunity to the N- and C-lobes of the protein to come close to each other, thus leading to a conformation change.

### 3.3. Effect of Change in Temperature: More Dynamics of the Protein

#### 3.3.1. Residues 34–110 Distance Distribution

**Figure 6:**
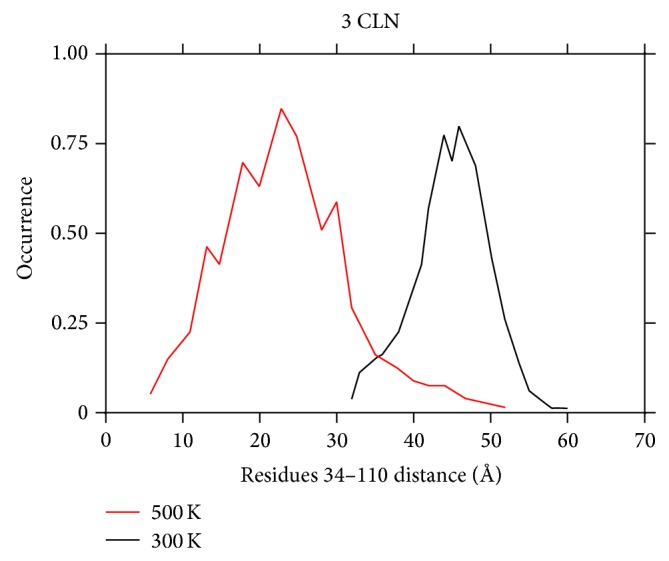
The normalized distance distribution (in Å) measured between residues 34 and 110, observed at the end of 100 ns in the 3CLN run at 300 and 500 K. The ionic strength corresponds to the physiological value in both simulations.

Here, the behavior of CaM is observed at a temperature of 300 and 500 K at physiological pH value. With an increase in the temperature, the protein is observed to acquire a more compact form as implied from the normalized distance distribution in Figure 6. The distance distribution here is observed to shift from a mean value of 48 Å to 26 Å with a change in the temperature to 500 K from an initial value of 300 K. This implies that an increase in the temperature helps the N- and C-lobes to come close to each other. This change is in accordance with the previous case where a change in the ionic strength also paves the pathway for the two lobes to come close to each other.

#### 3.3.2. Root Mean Square Deviations (RMSD)

**Figure 7:**
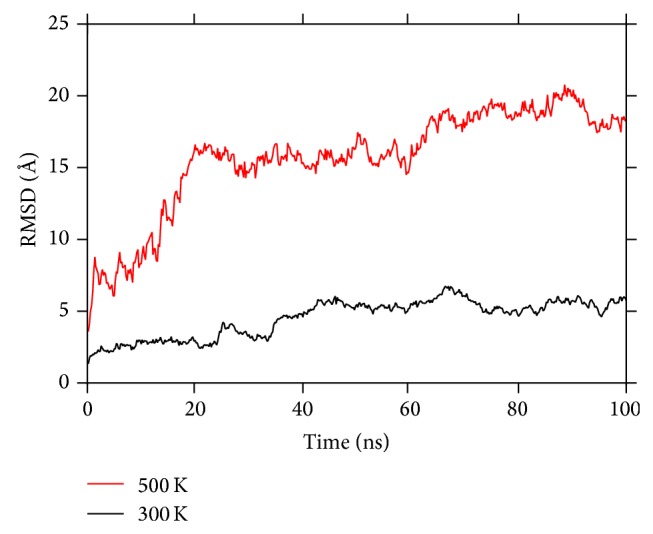
RMSD of the protein as a function of time at different temperatures. A significant deviation can be observed at 500 K as compared to 300 K implying a much greater flexibility at 500 K.

The RMSD value of the overall protein structure corresponding to both the temperature cases is compared as seen in Figure 7. The average value of the RMSD increases from 5 Å to 18 Å on increasing the temperature from 300 K to 500 K. Thus a significant shift in the average value of the RMSD is observed with an increase in the temperature. This increase in the RMSD value would be the result of kinetic energy imparted to the protein residues at a higher temperature. The kinetic energy imparted to the protein would affect the stability of the protein to a much greater extent and confirms the previous observation of the change in the conformation on changing the temperature.

#### 3.3.3. Salt Bridge Formation between the Residues of N- and C-Lobes

**Figure 8:**
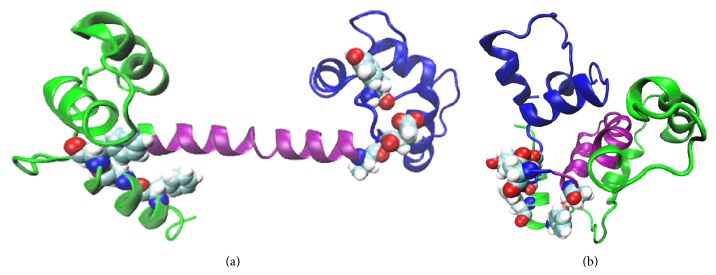
Snapshots from the MD simulation: N-lobe is in green, linker is in purple, and C-lobe is in cyan. At (a) 0 ns, that is, at the beginning of the simulation, (b) a salt bridge is formed between residues 11 (GLU) and 94 (LYS), 14 (GLU) and 90 (ARG), and 7 (GLU) and 86 (ARG) of the N-lobe and the C-lobe. Note that only the salt bridge formations are shown here and the calcium ions are not shown in these snapshots.

To further understand the dynamics and the conformation change occurring with an increase in the temperature, we monitor all possible interactions between the residues of the two lobes of the protein. These interactions are studied in terms of the salt bridge formations between the residues of the two lobes. A number of salt bridges are observed to be formed between the residues of the N- and C-lobes of the protein. Three salt bridges are observed to be formed between the residues 11 (GLU) and 94 (LYS), 14 (GLU) and 90 (ARG), and 7 (GLU) and 86 (ARG) between the N-lobe and the C-lobe as seen in Figure 8. These three salt bridges once formed are observed to be present for the rest of the simulation and thus lead to a more compact form of the protein. The linker is also observed to bend at the time of these salt bridge formations thus leading to a more compact structure. This is consistent with the earlier work where flexibility of the linker was assumed to be the main cause of the conformation change of CaM [28–30].

In order to statistically validate the data, another set of simulations is performed for 100 ns starting with 3CLN at an increased temperature of 500 K. A conformation change is observed with the formation of salt bridges between the residues of N- and C-lobes. The distance distribution is also observed to shift to a lower end thus leading to compact form of the protein. The data is not shown here due to the brevity of space.

## 4. Interaction Energy between the Two Lobes

**Figure 9:**
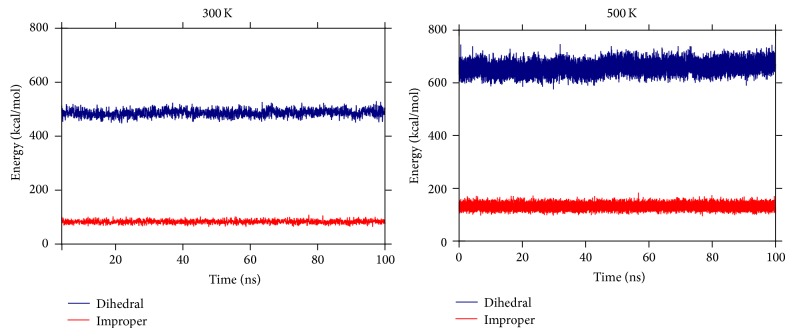
The improper and dihedral parts of the interaction energy contributions at 300 K and 500 K are shown here. These two parts of the total energy are observed to play a key role in the conformational change of the protein.

To further understand the dynamics of the protein at a higher temperature in detail, we perform an energy calculation using the “NAMD energy” plugin in the VMD program as in [15, 16]. This plugin calculates the various interaction energy terms between the different parts of the protein. The interaction energies between the N- and the C-lobes of the protein would help to understand the conformational changes happening on changing the different environmental conditions. The overall energy of the protein is not observed to show any significant shift on changing the temperature to a higher value. Only the improper and dihedral terms of the total energy are observed to show a significant shift on increasing the temperature to 500 K as seen in Figure 9. The NAMD energy plugin calculates these energy contributions by taking into account all the improper and dihedral terms of the protein. This significant shift in the improper and dihedral energy terms thus indicates that the two lobes of the protein come close to each other on changing the temperature to a higher value and thus lead to a conformation change. Other parts of the total energy of the protein such as kinetic and potential do not show any significant contribution in this conformation change. Also, no hydrophobic patch formation is observed in this case in contrast to what is observed in [15, 16] where a hydrophobic patch was observed to add an entropic contribution to the free energy. This patch thus compensates for the increase in the interaction energy of the two lobes. Solvation energy on the other hand can be ignored in this case as here we are only interested in the terms leading to a significant contribution to the conformation change of the protein. This conformation change would be better described in terms of its bonds, angles, and dihedrals.

## 5. Discussion

The environmental conditions would play the most important part in the functioning of the various proteins. The role of calcium ion removal, change in ionic strength, and variations in the temperature in altering the conformation distributions as well as the dynamics of Ca2+-CaM is studied using extensive MD simulations starting with the extended form of the CaM protein (3CLN). This gave a list of possible alterations in the environmental conditions of the protein which could pave the pathways for its conformational changes and/or add flexibility to it. Though the complete list of these environmental conditions would be more extensive, in this work, we explore variations in three environmental conditions as a first step.

Changing the ionic strength and changing temperature are observed to be the key factors playing significant role in the conformation change of the calmodulin protein. A significant shift in the normalized cumulative distance distribution of the residues numbers 34–110 is observed at a lower ionic strength of 150 mM. This cumulative distance distribution is observed to shift from a peak of 48 Å to a value of 30 Å on changing the ionic strength to 150 mM implying a much compact structure of the protein. This shift in the distance distribution occurs due to the loss of electrostatic interactions at a lower ionic strength of the solution because the lesser concentration of the salt ions elucidates the screening of the salt ions on solute. Finally it gives a good opportunity to the two lobes to come close to each other, thus leading to a conformation change of the protein.

On the other hand, the distance distribution between the residues 34 and 110 of the protein does not show a significant shift on the removal of the calcium ions from the two lobes. Only a slight shift in the RMSD value of the protein is observed for different stages of the calcium ion removal which signifies only enhancement in the flexibility of the protein. Thus calcium ion removal just acts as an attribute to add flexibility to the overall protein rather than giving any overall compactness to the structure. The temperature and pH are kept at 300 K and physiological value of 7.4 in both the above-mentioned cases.

On changing the temperature to a higher value of 500 K, the salt bridge formation between the N- and C-lobes leads to an overall lowering of the energy of the protein. Three salt bridges are observed to be formed between the residues 11 (GLU) and 94 (LYS), 14 (GLU) and 90 (ARG), and 7 (GLU) and 86 (ARG) of the N- and the C-lobes. These salt bridges once formed are observed to be present throughout the simulation. This favors the bending of the two lobes towards each other and thus leads to a more compact form as compared to the initial extended structure. Also the improper and the dihedral terms of the total energy show a significant shift on increasing the temperature. The normalized distance distribution between the residues numbers 34 and 110 is also observed to shift from an initial value of 48 Å to 26 Å in this case, which validates a conformation change in this case. A repetitive set of simulations is also performed to check the reproducibility of the results in this case.

Thus an important set of possible alterations in the environmental conditions of CaM protein are studied which could lead to a conformation change in the protein. Two of the conditions studied are observed to give rise to a conformation change whereas one just adds flexibility to the overall protein structure. A detailed study on the variation of these conditions is under progress.

## 6. Summary

Among the numerous proteins present inside our body, calmodulin (CaM) is one of the most important proteins which plays a very crucial role in the metabolism and physiology of the eukaryotes. CaM mediates processes such as inflammation, metabolism, apoptosis, muscle contraction, intracellular movement, short-term and long-term memory, nerve growth, and the immune response. CaM is a calcium-binding protein that can bind to and regulate a multitude of different protein targets, thereby affecting many different cellular functions. As it can bind to a number of protein targets, its metabolic activities would greatly be affected by the environment it is present in. Therefore, it is interesting to study the effect of changing the different environmental conditions on this protein. In this work we have computationally predicted the conformation changes that would be possible on changing the environmental conditions such as ionic strength, calcium ion concentration, and the temperature. We observed that a change in ionic strength and temperature would give rise to a conformation change of the protein whereas calcium ion removal just acts as attribute to add flexibility to the overall protein structure. Also we get useful physical insights of the events which give rise to these observed conformation changes.
